# Manual therapies in cystic fibrosis care: a scoping review

**DOI:** 10.1186/s12998-023-00478-0

**Published:** 2023-02-06

**Authors:** Niklas Sposato Sinderholm, Kristofer Bjerså

**Affiliations:** 1 Department of Health and Rehabilitation, Institute of Neuroscience and Physiology, Sahlgrenska Academy, University of Gothenburg, 413 45 Gothenburg, Sweden; 2 Department of Surgery, Clinical Sciences, Sahlgrenska Academy, University of Gothenburg, 413 45 Gothenburg, Sweden

**Keywords:** Cystic fibrosis, Pain, Muscle strength, Respiration, Manual therapies, Musculoskeletal manipulation

## Abstract

**Objectives:**

To review the use of manual therapies (MT) for pain, respiratory muscle strength and pulmonary function in cystic fibrosis (CF) care.

**Methods:**

A search with a systematic approach was conducted by two independent reviewers, using the databases Medline, PubMed, Scopus and Cinahl from their respective inception dates to March 2021.

**Results:**

A total of 199 publications were initially screened by title and abstract, after which 190 were excluded. Following a full-text review of the remaining articles, six studies with a total of 234 participants were included. Decreased pain levels following MT were observed in two studies and, in three studies, patient reports on improvement in ease of breathing and peak airflow were presented. No significant effects on spirometry measures were observed and none of the included studies investigated respiratory muscle strength.

**Conclusion:**

Current research on MT in CF care indicates positive trends based on subjective measures. However, research in this context is sparse and disparate in terms of both interventions and methodology. Further investigations including MT as part of multimodal interventions are therefore suggested before any specific recommendations for clinical implementation of MT in CF can be provided.

**Supplementary Information:**

The online version contains supplementary material available at 10.1186/s12998-023-00478-0.

## Main text

Cystic fibrosis (CF) is a serious inherited multi-organ disease, primarily affecting the lungs and the gastrointestinal system [1, 2]. The incidence of CF is calculated to be 1 in 3000 live births [3]. Following the discovery of the cystic fibrosis transmembrane conductance regulator (CFTR) gene in 1989, significant medical advances have been made. These advances have since led to a considerable increase in median life expectancy, at present of close to 50 years [4]. As patients diagnosed with CF today are expected to reach adulthood, new biomedical, psycho-emotional and social challenges have arisen [5, 6]. Subsequently, care strategies and research perspectives need to be broadened to allow for individualisation and greater patient independence in accordance with the patient’s distinct medical needs, as well as contextual circumstances.

Interventions for patients with CF vary depending on the disease process but consistently include combinations of pharmacological treatments and different treatment modalities included in physiotherapy [7]. For CF patients with critical conditions, surgery might also become a necessary life-saving route [7–10]. However, the explorative purpose of this review resides within the context of manual therapies and physiotherapy.

Physiotherapy interventions in CF care have developed over time, from an initial main emphasis on airway clearance, including manual chest percussions, to a broader scope of practice, which today also includes personalised exercise programmes, daily activity, and self-care plans [11–13]. These daily routines are time- consuming and result in continuous strain on the musculoskeletal elements of the respiratory system. The consequences of these physical demands have been presented in previous research as pain and stiffness within the thoracic and spinal regions [11, 14, 15].

One group of interventions, used in the treatment of musculoskeletal pain and dysfunction, are manual therapies (MTs). MTs as part of conventional care have mainly been explored by chiropractors, osteopaths and physiotherapist for various health care contexts and have been shown to have positive effects in several conditions, e.g., musculoskeletal pain, impaired mobility and respiratory muscle strength, anxiety, and gastrointestinal function [16–21]. In accordance with a broadened multimodal practice in CF, as described above, there is room to explore interventions that are currently not included in the international guidelines, e.g., MT. For the time being, there is a knowledge gap concerning the usefulness of MT in CF care. Hence, the aim of this study was to review published data on any form of manual therapy intervention aimed at affecting thorax associated musculoskeletal pain, respiratory muscle strength and pulmonary function in patients with cystic fibrosis.

This study was performed as a scoping review with a systematic approach in accordance with the Preferred Reporting Items for Systematic reviews and Meta-Analyses extension for Scoping Reviews (PRISMA-ScR) Checklist (attached in Additional file 1). Registration was carried out prospectively with PROSPERO; Registration number CRD42021233230.

**Fig. 1:**
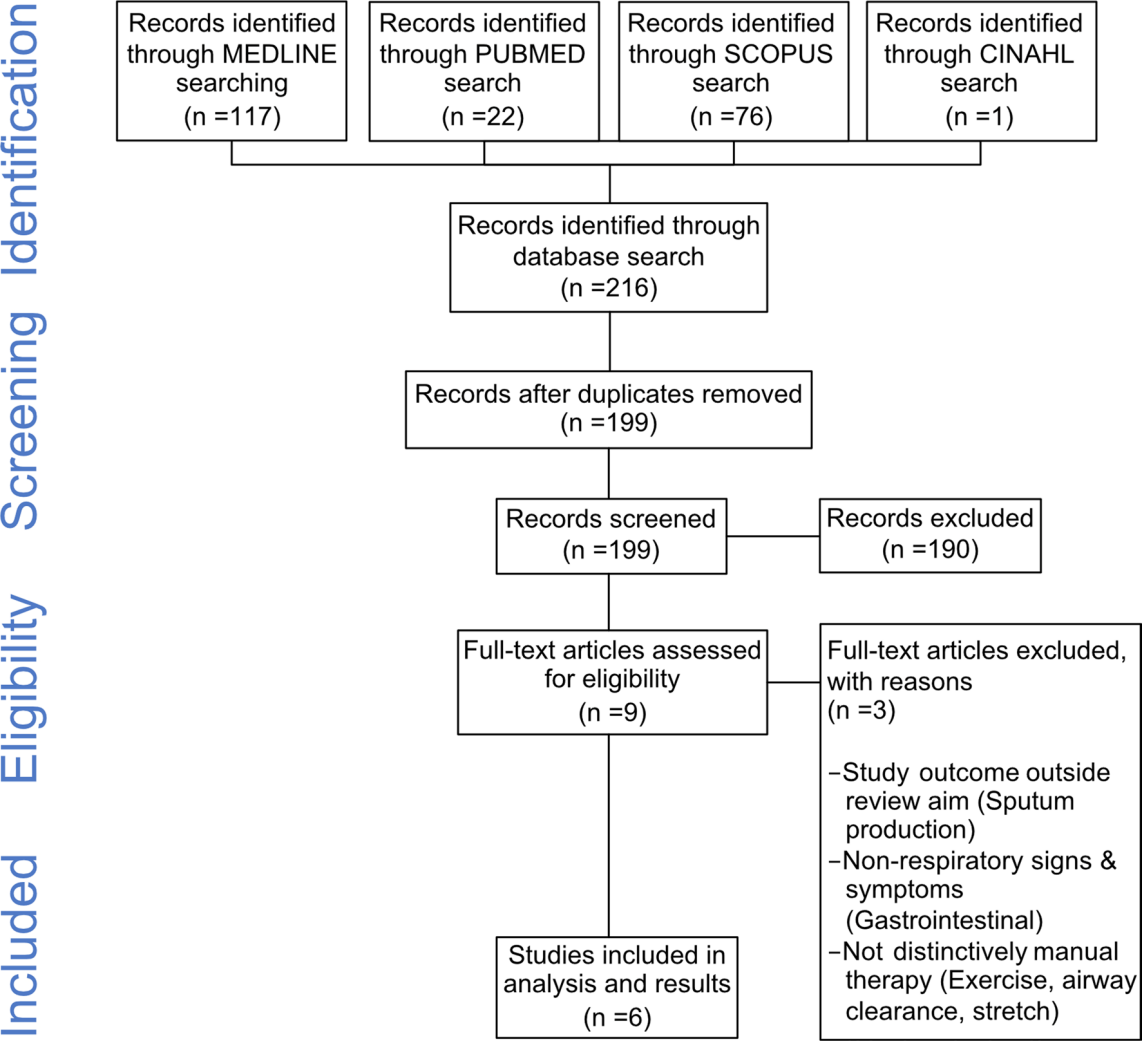
PRISMA flowchart of database search

A literature search was conducted using the databases Medline, PubMed, Scopus and Cinahl from their respective inception dates to March 2021. The search strategy was developed and applied in close collaboration with specialist librarians at the biomedical library at the University of Gothenburg. All retrieved publications were exported to EndNote X9.2 and duplicates removed, resulting in a total of 199 unique publications, see Fig. 1. A conference paper investigating osteopathic treatment of pain in patients with cystic fibrosis was found and assessed [22]. This paper was ultimately excluded on account of its condensed format, which limited the possibility for proper analysis, thereby rendering the paper unsuitable for this review. No additional publications outside the records identified through the database search were identified.

There was no involvement of patients or the public in this study. Inclusion criteria included all peer-reviewed research studies registered within stated databases, written in English, that included a study population of patients with cystic fibrosis and an intervention classified as MT. All studies meeting the inclusion criteria published prior to mars 2021 were included. The exclusion criteria were: Systematic reviews or other forms of review articles; Meta-analysis or meta-synthesis articles; Airway clearance percussive techniques using devices or performed manually; Studies or data focusing on gastrointestinal function outcomes in cystic fibrosis. The process and subsequent inclusions based on the set criteria are presented in Fig. 1.

Concerning data collection, titles and abstracts of retrieved articles were individually assessed by both authors (NSS and KB) based on the study aim and the set inclusion and exclusion criteria. This resulted in a matching outcome of six articles with no discrepancies between the two reviewers’ individual assessments. Data from the included articles were extracted separately by both authors within the three areas of pain, respiratory muscle strength and pulmonary function. In addition to this, data were extracted concerning study population demographics, applied therapies, intervention performing therapists, and reports of adverse effects. All included studies were graded based on the Oxford Centre for Evidence-Based Medicine 2011 Levels of Evidence.

Based on the small number of articles as well as the low evidence level within the included studies, a narrative synthesis based on the extracted data was made to present the investigated outcome measures. Meta-analysis methodology as well as other statistical summative calculations could not be performed due to the diversity of the used methodology, interventions, and outcome measures.

Concerning ethical approval and consent to participate, of the six included studies, four stated approval by an ethical review board [23–26]. Two studies did not include such statements in the article texts [27, 28].

Ethical approval and consent to participate with regards to the present scoping review was not applicable.

From the initial 199 unique publications in the database search, 190 were excluded after title and abstract screening. Of the remaining nine articles, six were included after a full-text review (see Fig. 1), resulting in two randomised controlled trials (RCTs), three pilot RCTs, and one consecutive case series. A demographical summary of the included articles is presented in Table 1. In total, 234 participants were included within the six articles, between the years 1999 and 2019, and within the healthcare systems in the United States, United Kingdom, France, and Australia. Investigated manual therapy interventions are presented in Table 2.

**Table 1:** Demographics

Authors	Year of publication	Journal	Country of data collection	Study design	N of participants	Participant age (mean; min-max)	Participant gender distribution (W/M)	Outcome measures
Hernandez-Reif, M Field, T Krasnegor, J Martinez, E Schwartzman, M Mavunda, K	1999	Journal of Pediatric Psychology	USA	RCT	20	9,9 (5-15)	NA	*Pulmonary function* Peak air flow *Anxiety and mood* STAI, STAIC, POMS
Lee, A Holdsworth, M Holland, A Button, B	2009	Journal of Cystic Fibrosis	Australia	Consecutive case series	105	30,5 (NA)	NA	*Pain* Manual pain assessment, Musculoskeletal pain level by VAS *Pulmonary function* Ease of breathing by VAS
Sandsund, CA Roughton, M Hodson, ME Pryor, JA	2011	Physiotherapy	UK	Single-blinded Pilot RCT	20	27 (25-34)	50/50%	*Pain* Chest wall pain level by VAS *Pulmonary function* FEV_1_, Quality of Life: CFQoL section 1 *Other* Thoracic index, Chest wall excursion, Modified shuttle test
Hubert, D Soubeiran, L Gourmelon, F Grenet, D Serreau, R Perrodeau, E Zegarra-Parodi, R Boutron, I	2014	PLOS One	France	Double-blinded Pilot RCT	32	34 (NA)	81.2/18.8%	*Pain* Daily pain diary by VAS with focus on chest and back, Neck/Trapezius pain, Headache, Analgesic consumption. *Quality of Life* CFQoL
Swender, DA Thompson, G Schneider, K McCoy, K Patel, A	2014	The Journal of the American Osteopathic Association (JAOA)	USA	Single-blinded RCT	33	24,5 (18-50)	42.5/57.5%	*Pain* Change of general pain on a three-level scale *Pulmonary function* FEV_1_%, Spirometry, Change of breathing quality on a three-level scale. *Anxiety and mood* Change of general anxiety on a three-level scale. *Other* Weight, temperature, heart rate, pulse oximetry, blood pressure, respiratory rate
Zink, K Chini, B Cowens, J Kremer, L Lin, L	2019	International Journal of Therapeutic Massage and Bodywork	USA	Pilot RCT	24	15,7 (9-20)	67/33%	*Pain* Musculoskeletal pain by NRS11 *Pulmonary function* Spirometry, Ease of breathing, Thoracic excursion *Quality* *of Life* PedsQL, CFQ-R, author created three item NRS

**Table 2:** Interventions and control group interventions

	Intervention providers	No. of unique intervention providers	No. of treatment sessions	Duration of each treatment session (min)	Specified manual therapy interventions*	Control group intervention
Hernandez-Reif et al. [28]	Parents	20	30	20	Massage (Clearly defined full body massage with relaxing intent)	20 min reading
Lee et al. [24]	Physiotherapist	NA	1	60	Joint mobilization, intercostal mobilization, soft tissue therapy and remedial massage. (Specific treatment protocol not clearly disclosed)	None/Standard care
Sandsund et al. [25]	Physiotherapist	1	6	45	Specific mobilisations to the rib cage and thoracic spine, Trigger point treatment (Treatment methodology described on the basis of referenced literature)	None/Standard care
Hubert et al. [26]	Osteopathic practitioner	1	6	60	Osteopathic manipulative treatment (OMT): Cranial techniques, Muscle techniques (static and dynamic soft tissue manipulation), Structural techniques (Passive joint mobilization with and without impulse), Visceral techniques (Myofascial techniques addressing respiratory and abdominal structure and function) (Methodology and protocol clearly stated in attachment)	Clearly specified Sham OMT and Standard care
Swender et al. [23]	Osteopathic physicians	3	4–7	15	Rib raising (passive low velocity joint mob). Abdominal diaphragm release (Soft tissue traction). Thoracic inlet myofascial release (Soft tissue traction). Thoracic lymphatic pump (Rhythmic mobilisation) Suboccipital decompression (Myofascial pressure and traction)	Clearly specified Sham OMT
Zink et al. [27]	Licenced massage therapists	4	3–5	60–90	Deep tissue massage, Myofascial Triggerpoint treatment (MTrP) and active stretch (PIMR) full body but with an emphasis on respiratory muscles (Treatment protocol clearly described)	None/Standard care

*As termed in the respective articles with reviewers comment and interpretation in brackets

The first objective of the results was measures of pain, which were included in five studies [23–27], by using a wide range of instruments. The use and form of MT interventions for pain varied considerably among the included studies. In a study including both CF stable patients and patients with an acute CF exacerbation, statistically significant (p > 0.001) improvement was indicated with a mean difference of 18 mm by visual analogue scale (VAS), prior to and following intervention [24]. MT intervention included joint mobilisation, soft tissue manipulation and massage. A trend of decreased pain levels in the treatment group as compared to the control group, measured on a numerical rating scale (NRS), was also indicated in a pilot RCT study investigating the effects of massage therapy on pain in children and young adults [27]. In contrast, findings following osteopathic manipulative treatment (OMT) in a single blinded RCT did not determine either statistical or clinical significance between the treatment and control groups, measured by NRS [23]. These results are in accordance with two RCT studies that measured pain by VAS which also found no statistical difference between intervention and control groups following joint and muscular mobilisation in a physiotherapy and an OMT setting respectively [25, 26].

Further in the results, none of the included studies in this review investigated the effects of MT on respiratory muscle strength in CF.

Next, qualitative and quantitative outcome measures of pulmonary function were included in five studies of the results [23–25, 27, 28]. In an RCT that included patients admitted to hospital due to exacerbation, nearly all participants in the intervention group reported ease of breathing (EOB) following OMT [23]. In contrast, less than1/3 of the patients that received sham treatment reported any positive change in EOB. These measures where extracted from a five-item questionnaire. Comparably, another study showed general improvement in EOB in both clinically stable patients, as well as in patients with acute exacerbation, as measured by VAS [24]. In addition, the results from the acute exacerbation group showed statistically significant (p > 0.001) improvement with a mean difference of 5 mm in VAS. Treatment consisted of a single massage and spinal joint mobilisation session. Furthermore, an RCT sought to explore the potential benefits of massage therapy in children with generally mild CF and their parents [28]. One of the study’s primary outcomes, peak airflow, indicated a positive trend towards improvement in the treatment group. However, a statistical comparison between treatment and control was not presented. Forced expiratory volume in one second (FEV1) was measured and compared between the intervention and control groups in three studies [23, 25, 27], of which one study also measured forced vital capacity (FVC) [27]. No statistical differences were found between the intervention and control groups in any of these studies concerning measures of FEV1 or FVC.

Finally in the results, out of the six included studies, three investigated negative side effects in relation to their respective studies [23, 25, 26]. Of these, mild nausea following OMT was reported by one participant in one study [23], whereas no side effects were reported by participants in the remaining two [25, 26].

Based on these results, a number of issues need to be discussed. Given its objectives, this scoping review indicates that research on MT, as part of CF care is currently limited and heterogeneous in terms of outcomes and interventions. The severity of the disease, which historically has necessitated a primary research focus on pathophysiology, pharmacology, and surgery, might to some extent explain this. Still, different treatments with a general objective to promote physical activity and distinctively as airway clearance treatment have been, and remain, an essential part of CF care [13, 29–33]. However, MT as presented in the studies included in this review has rarely been part of CF care. As such, statements concerning implications for clinical practice or education at this stage would be highly speculative. On the other hand, the results from this study show several ways to proceed with clinical investigations in the future. Given the severity of the disease and the already high burden of care for patients with CF, it is reasonable to suggest carefully conducted pilot studies before full-scale multicenter studies are carried through.

Within this study, three main areas were investigated: pain, respiratory muscle strength and pulmonary function. Concerning pain, several previous studies have presented that both children and adults with CF suffer from pain symptoms derived from the chest and abdomen, but also from several other bodily areas [34, 35]. In addition, it has been found that elevated pain levels among patients with CF are associated with increased exacerbation frequencies and decreased quality of life [36]. A need for better pain management has been stated [35], and from the results of this study more research is needed before MT can be considered as an intervention itself or as part of multimodal care strategies. Furthermore, the complexity of pain in general has been extensively researched and consequently, its multi-layered mechanisms are broadly understood in most contexts. Although certain elements of thoracic pain, i.e. radicular and facet joint pain, and the relation of thoracic pain to MT have been previously determined [16], musculoskeletal thoracic-related pain has remained under-investigated [37, 38].

The level at which respiratory muscles can generate force is dependent on mechanical and functional properties, e.g., the shape and angulation of the ribs, thoracic and spinal mobility, muscle fibre length and muscle fibre type [39–43]. As one or more of these properties can be changed by pulmonary disease processes, muscle strength can become negatively affected. MT has been shown to have positive effects on respiratory muscle strength [20, 21]. However, none of the studies included in this review reported on the effects of MT on respiratory muscle strength in CF. It is thus suggested that future research should include such investigations as well as exploration of structural adaptive changes in patients with CF.

Loss of thoracic mobility negatively affects respiratory pump function [44]. Conversely, MT, with an aim to increase mobility in both healthy individuals and in patients with respiratory disease, has in previous research shown positive short-term effects on pulmonary functions [20, 45–48]. Within these previous studies both subjective measures as well as objective outcomes indicated encouraging results. In contrast, the results from the studies included in the current review indicated significantly positive outcomes solely regarding subjective measures. This discrepancy may be due to differences in pathophysiological processes between CF and other respiratory diseases, or to methodological limitations, including sample size and interventions.

The current top priority, as determined by patients, caregivers and health care providers within CF, is to reduce the treatment burden [49]. To date, research has not fully responded to this priority. This review has shown that there is very limited research on the therapeutic benefits of MT as an integrated part of CF care. However, there are indications of functional benefits and pain-relieving effects. MT as stand-alone interventions and/or as part of multimodal care need to be investigated further, particularly in reference to the MT modality, dosage, and proposed mechanisms.

## Conclusion

Manual therapies within the area of cystic fibrosis care have been investigated only to a limited extent, and although current research indicates positive trends based on subjective measures, i.e., pain and ease of breathing, research in this context is disparate in terms of both interventions as well as methodology. Based on the increased life expectancy among patients with CF, and those new challenges that have come as a result of this positive development, we suggest a wider scope for future research. Such investigations could include MT as part of multimodal interventions, addressing thoracic pain and functional properties of respiration in CF care.

## Supplementary Information

**Additional file 1**. S1 - PRISMA checklist.

## Data Availability

Data will be available upon request.
